# Primary Malignant Lymphoma Presenting as an Isolated Epidural Mass

**DOI:** 10.3390/diagnostics12102462

**Published:** 2022-10-11

**Authors:** Haksung Kim, Daekyun Kim, Seokwon Kim

**Affiliations:** Department of Neurosurgery, College of Medicine, Chosun University, Gwangju 61453, Korea

**Keywords:** lymphoma, epidural mass, spine

## Abstract

Primary central nervous system lymphoma is a rare form of extranodal non-Hodgkin’s lymphoma that occurs in the brain, spinal cord, leptomeninges, or eyes and typically remains confined to the central nervous system. Among them, malignant lymphoma presenting as a primary tumor of the spinal cord is extremely uncommon, and epidural mass formation is known to occur in only 0.8–2.8% of cases of malignant lymphomas. Furthermore, primary malignant lymphoma presenting as an isolated epidural mass is much rarer. Here, we report a case of primary malignant lymphoma of the thoracic spine presenting as an isolated epidural mass that did not involve the vertebral body or posterior element. Surgical decompression is essential to prevent further neurological deterioration. Here, we present a successful treatment strategy for this rare case.

A previously healthy 53-year-old male patient was admitted to our emergency room (ER) for the progressive weakness of the lower extremities with urinary incontinence. The patient did not complain of fever, night sweats, or progressive weight loss. Physical examination revealed hypoesthesia on the anterior aspect of both thighs with a decreased muscular strength of both lower extremities (grade III). However, he was able to straighten or raise his legs. Infection markers, including erythrocyte sedimentation rate and C-reactive protein, were within normal limits. Magnetic resonance (MR) imaging of the thoracolumbar spine revealed an epidural mass compressing the spinal cord from T7 to T9 (Figure 1). The epidural mass showed homogeneous enhancing signal intensity on contrast-enhanced MR images. The patient underwent emergency decompressive laminectomy and mass removal. Intraoperatively, a dark, grayish, and avascular epidural mass was observed and removed for cord decompression and histopathological examination. The mass did not adhere to the dural sac and was easily removed by CUSA. Histopathological analysis of the specimen revealed malignant lymphoma of the diffuse large B-cell type.

Immunohistochemical characterization showed that the results of tumor testing were positive for B-cell marker CD20 and negative for T-cell marker CD3 and the epithelial marker cytokeratin (Figure 2). Further evaluations were performed based on the indicated systemic lymphoma characteristics. MR imaging of the brain and cervical spine were normal. Further work-up, including computed tomography (CT) of the thorax and abdomen and ultrasound of the lymph nodes, showed systemic disease.

Postoperatively, significant pain relief was achieved, and the patient was transferred to the hematology and oncology medical department for chemotherapy with radiotherapy. He underwent four cycles of chemotherapy followed by radiotherapy, and 40 Gy was administered in 20 fractions. The patient was pain-free and able to return to full-time work thirteen months after surgery.

The term lymphoma is used when the process is confined to a mass lesion with minimal or no evidence of peripheral blood and bone marrow involvement [1,2,3]. Isolated primary malignant lymphoma is a rare form of extranodal lymphoma, and severe compression of the spinal cord as the initial manifestation of primary malignant lymphoma of the spine is much rarer, with an incidence of less than 5% [4,5,6]. It is known to occur mainly in the thoracic region. Our patient initially presented with severe cord compression not involving osseous lesions such as the vertebral body or posterior element. The mass was primarily located in the posterior epidural space. Although the exact mechanism underlying isolated epidural mass formation without osseous involvement or systemic lymphoma has not been clarified, the mass is thought to originate from the bone marrow [7]. A small number of lymphocytic cells can migrate to the spinal epidural space directly through the Haversian canals of the vertebral bone hematogenously via the epidural venous plexus, followed by mass formation even before systemic lymphoma [8].

Primary malignant lymphomas of the spine are often difficult to diagnose and confused with epidural hematoma or other spinal tumors, especially malignant metastases. They are usually isointense to hypointense on T1-weighted MR images and isointense to hypointense on T2-weighted images with contrast enhancement [9]. However, CT and MR provide only indirect diagnostic evidence, and the true diagnosis can only be made based on histopathological examination. The treatment of these lesions is multifactorial, and it includes decompressive surgery, radiotherapy, and chemotherapy. Liu et al. reported that well-established treatments, such as chemotherapy and radiotherapy or combined treatments, are a mainstay, and surgery should be performed in selected cases [10]. However, early surgical decompression and mass removal are mandatory in cases of significant neurological deficits [7]. Owing to the manifestations of spinal cord compression, emergent surgical decompression with a pathological diagnosis is essential for a favorable outcome.

We report a rare case of a primary malignant lymphoma that developed as an isolated epidural mass. This case demonstrates that spinal surgeons should consider primary malignant lymphoma in the differential diagnosis of an isolated spinal epidural mass, especially in the thoracic spine.

## Figures and Tables

**Figure 1:**
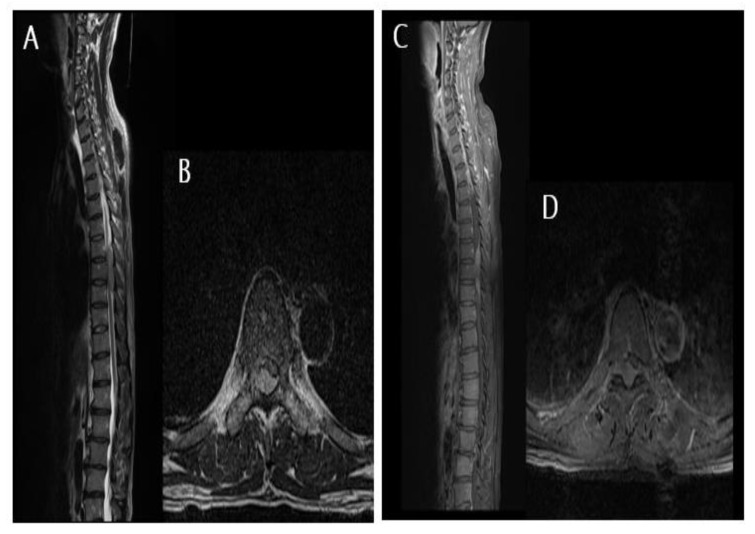
Magnetic resonance images of the patient. (**A**,**B**) T2-weighted sagittal and axial images showing an epidural mass lesion compressing the spinal cord from T7 to T9. (**C**,**D**) Contrast-enhanced magnetic resonance images reveal homogeneous enhancing epidural lesions at T7–T9 levels.

**Figure 2:**
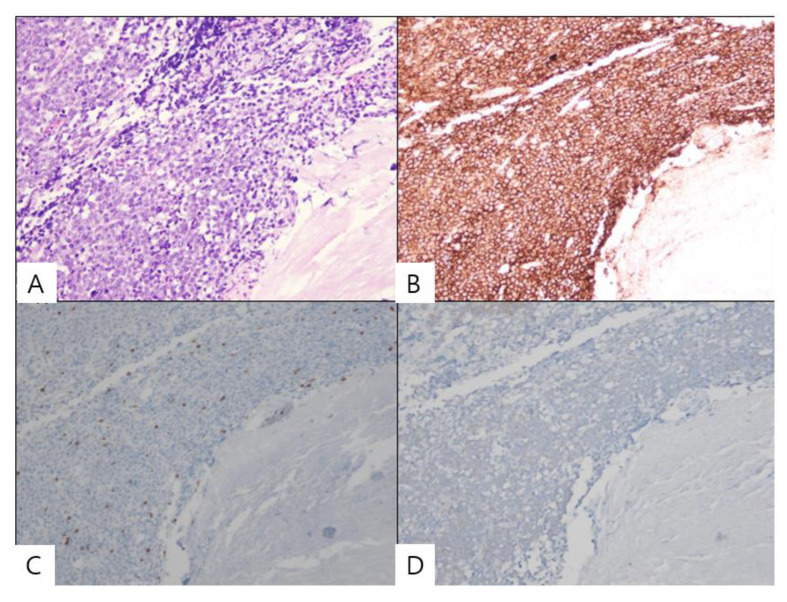
Histopathological examination findings of the patient. (**A**) Microscopically, the diffuse proliferation of atypical lymphoid cells is identified. (**B**) The tumor cells are immunoreactive for the B-cell marker CD20. (**C**,**D**) However, the tumor cells are not immunoreactive (X40) for the T-cell marker CD3 and the epithelial marker cytokeratin.

## Data Availability

Not applicable.
